# Examining longitudinal changes in visuospatial working memory in adolescents with Developmental Language Disorder

**DOI:** 10.7717/peerj.21177

**Published:** 2026-04-23

**Authors:** Jorge-Luis Guirado-Moreno, Raúl López-Penadés, Àngels Esteller-Cano, Elma Blom, Eva Aguilar-Mediavilla, Daniel Adrover-Roig

**Affiliations:** 1 Investigació en Desenvolupament, Educació i Llenguatge (I+DEL), Institut de Recerca i Innovació Educativa (IRIE), Palma, Illes Balears, Spain; 2 Department of Development and Education of youth in Diverse Societies (DEEDS), Utrecht University, Utrecht, Netherlands

**Keywords:** Developmental language disorder, Visuospatial working memory, Adolescents, Cognitive development, Updating

## Abstract

**Introduction:**

Adolescents with Developmental Language Disorder (DLD) exhibit difficulties in language abilities, affecting their capacity to produce and/or comprehend spoken language. In addition to a main language deficit, DLD is often associated with impairments in executive functioning (EF), including working memory (WM).

**Method:**

A longitudinal study was conducted with 38 participants: 12 adolescents with DLD and 26 typical development (TD) peers. Visuospatial WM was assessed using the Backward Corsi Task (BCT) at three time points.

**Results:**

Adolescents with DLD consistently scored lower than TD on the BCT across all three waves. A significant main effect of wave was also found, indicating that performance improved over time. However, improvements were only significant from Wave 1 to Wave 3, and not between adjacent waves. No significant Group-by-Wave interaction was observed, suggesting similar developmental trajectories across groups.

**Conclusions:**

The findings suggest that while adolescents with DLD show improvements in visuospatial WM over time, their performance seems to remain consistently below that of their TD peers. This may indicate that visuospatial WM difficulties associated with DLD persist into adolescence. These results might be partly explained by the task’s engagement of the central executive component, as tasks with high executive demands could account for the observed group differences.

## Introduction

Children and adolescents with Developmental Language Disorder (DLD) exhibit difficulties in language abilities, affecting their capacity to produce and comprehend spoken and written language. These difficulties may involve the expressive dimension, the receptive dimension, or both, reflecting the heterogeneity of this condition (Iverson & Williams, 2025). The term DLD is used when the language disorder is not associated with a known biomedical etiology (Bishop et al., 2016, 2017). Although children with DLD have a primary deficit in language, they often show difficulties in executive functioning (EF) (Blom & Boerma, 2020; Pauls & Archibald, 2016).

EF refers broadly to higher-order goal-directed processes essential for situations where automatic responses or instincts are often inadequate, such as impulsive behaviors (Miyake et al., 2000). In this regard, the influential model originally proposed by Miyake et al. (2000) has been refined over the years (Friedman & Miyake, 2004, 2017; Miyake & Friedman, 2012), evolving into what is known as the “unity and diversity” framework of EF. According to this model, EF can be considered as a unitary construct in the sense that all executive processes share a common underlying factor—referred to as the Common EF—associated with maintaining goal-directed control. At the same time, EF encompasses partially distinct but interrelated components, such as inhibition, switching, and updating, which contribute uniquely to complex cognitive behavior. Using EF is effortful yet it is vital for psychological and physical health, success in school and life, and cognitive, social, and psychological development (Diamond, 2013).

One crucial component of EF is updating, which involves monitoring and encoding incoming information to assess its relevance to the current task and, lastly, revising outdated or irrelevant information with newer information. The fundamental aspect of updating involves actively manipulating pertinent information within working memory (WM), rather than simply storing it passively. According to Baddeley’s (2012) model of WM, the central executive system is responsible for the control and regulation of three subsystems: a phonological loop (specialized for the temporary storage and rehearsal of verbal and acoustic information), a visuospatial sketchpad (involved in the processing and temporary storage of visual and spatial aspects), and an episodic buffer (a limited-capacity system that temporarily stores and integrates information from multiple modalities into a unified, multimodal representation).

Previous literature examining the role of different components of WM on DLD found an association with limitations in verbal WM (see, for example, Bishop, North & Donlan, 1996; Larson & Weismer, 2022; Montgomery et al., 2019; Niu et al., 2024), whereas its relationship with the nonverbal domain is less clear. Although recent domain-general theories propose that the visuospatial WM from Baddeley’s (2012) model may also encompass information from other modalities, such as auditory or tactile (Morey, 2018; Poirier et al., 2019; Shipstead & Yonehiro, 2016), the literature has widely reported information from tasks that require the temporary storage, manipulation, and recall of visual and spatial information, such as remembering object locations or sequences of spatial positions (*e.g*., the Backward Block-Tapping task, Spatial Span task, Dot Matrix Backward task, Mr. X, nonverbal N-back task or Odd-One-Out task). In this line, the literature examining the role of visuospatial WM in DLD is less consistent (than that for verbal WM), particularly among children aged 6 to 12 (Arslan et al., 2020; Henry & Botting, 2017; Vugs et al., 2013). Taken together, it remains unclear whether the WM deficit observed in DLD is restricted to verbal abilities or whether it extends to visuospatial domains.

In general, studies examining visuospatial WM in *young* children (preschoolers) with DLD show that they present poorer performance than typical development (TD) peers, and that this is consistent across different measures. For example, a study centered on preschoolers (4–5 years old) and another on children aged 3 to 6 years showed that children with DLD scored significantly lower compared to their peers in a Backward Block-Tapping task (Everaert et al., 2023; Kapa, Plante & Doubleday, 2017). Similarly, Vugs et al. (2014) found that 4–5-year-old children with DLD scored lower than their TD peers on three distinct visuospatial WM tasks from the Automated Working Memory Assessment (Alloway, 2007) as well as on behavioral ratings (information from caregivers; BRIEF-P), indicating weaker abilities in this domain.

When examining *school-aged* children or older, results seem to be less consistent. Botting et al. (2013) assessed children aged 5 to 12 years on various tasks that required differential verbal involvement in WM tasks. Results indicated that children with DLD showed difficulty on all tasks with verbal elements but not in the Visual Block Recall task. Another study showed that children with DLD, who were on average between 7 and 8 years old, made more false alarms in a nonverbal N-back task, but on other visuospatial WM measures, they did not significantly differ from their TD peers (Lukács et al., 2016). In contrast, Weismer et al. (2017) did not find between-group differences on the nonverbal N-back task comparing children with DLD and TD who were on average between 9 and 10 years. Similarly, children with DLD (7–12 years old) performed similarly to their age-matched control peers in three visuospatial WM tasks: Odd One Out, Mr. X, and Spatial Span (Archibald & Gathercole, 2006). Finally, Acosta Rodríguez, Ramírez Santana & Hernández Expósito (2017) found that poorer performance in visuospatial WM of children with DLD (compared to TD children aged 5–11) was dependent on the severity of their language impairment. That is, only those who experienced impairment of the expressive *and* receptive dimensions of language showed difficulties in their visuospatial WM, while those with impairment only in the expressive dimension did not.

This overview suggests that age could be an important factor to consider. In line with this, some authors have proposed that visuospatial WM deficits could be part of the clinical profile of young children with DLD (Everaert et al., 2023), resulting in difficulties in visuospatial WM associated with specific developmental stages. In this regard, Arslan et al. (2020) explored verbal and visuospatial WM in children (7–11 years old) and adolescents (11–18 years old) with DLD. They reported that both children and adolescents with DLD, as compared to their age-matched peers, showed difficulties in verbal WM. In contrast, only children with DLD (and not adolescents) performed worse than TD in visuospatial WM, thus concluding that visuospatial WM deficits seemed to vanish during adolescence.

In addition to the separate studies discussed above, two meta-analyses have aggregated research findings on visuospatial WM in DLD, confirming the divergent results across ages. A recent meta-analysis that included 40 studies exploring differences in the performance of EF between DLD and TD children from 3 to 11 years old (Niu et al., 2024) revealed lower performance of children with DLD in two articles using the Backward Block Tapping task and four studies using the Spatial Span task. However, when analyzing age subgroups, the deficit in the Spatial Span task was observed only in the subgroup of preschoolers with DLD, but not in the school-aged subgroup (no studies included preschoolers for the Backward Block Tapping task, so no age subgroup analysis was conducted for this measure). By contrast, a meta-analysis that included 21 studies of children from 3 to 14 years old (Vugs et al., 2013) revealed that children with DLD performed significantly below their TD peers on visuospatial WM, and, interestingly, with no significant effect of age, as a continuous variable (as revealed by meta-regression analysis). Specifically, three studies in Vugs et al. (2013) show significantly poorer performance in visuospatial WM in children with DLD: Miller & Wagstaff (2011) observed significant differences in a Spatial Working Memory task with participants aged around 10 years; Karasinski & Ellis Weismer (2010) also found differences in the same task among adolescents (14 years); finally, Henry, Messer & Nash (2012) reported significant group differences in visuospatial WM at approximately 11.5 years using the Odd One Out task.

To clarify the potential effects of age on visuospatial WM deficits in DLD, longitudinal studies are relevant. In this vein, Vugs et al. (2017) evidenced that difficulties in visuospatial WM of children with DLD at 4–5 years old (Vugs et al., 2014) were no longer significant in the follow-up at 7–8 years when considering the mediating effects of age and nonverbal intelligence. Blom & Boerma (2020) conducted a longitudinal study with three time points, each separated by 1 year. The sample initially aged 5–6 years and approximately 8 years old in the final wave. Regarding visuospatial WM, measured with a Dot Matrix Backward task, children’s performance improved over time, with no significant differences between DLD and TD. In a second study with the same design but a larger group that included both monolingual and bilingual children, Boerma & Blom (2020) found that children with DLD performed overall worse than their TD peers, with both groups showing improved performance from the first to the third wave. However, when verbal short-term memory was controlled for, the difference between TD and DLD disappeared.

To our knowledge, apart from these three studies, no other research has investigated visuospatial WM in DLD longitudinally. In addition, research on this topic in older age groups, such as adolescents, is scarce and mixed, and no research has looked at visuospatial WM development in adolescents with DLD using a longitudinal design. The longitudinal design of the present study enables us to investigate visuospatial WM in adolescents with DLD throughout three waves of data collection, tracking their development between the ages of 11.5 and 14.5 years on average, and to contribute to this empirical gap. We hypothesized that (H1) the DLD group would perform poorer than the age-control group in the visuospatial WM task suggesting a relationship between DLD and visuospatial WM. Longitudinally, we anticipated (H2) an improvement in visuospatial WM scores over time in both groups, but particularly in adolescents with DLD, to the extent of matching the control group’s performance. This would suggest that visuospatial WM difficulties in DLD would only manifest in the early stages of development, but not during adolescence.

## Materials and Methods

### Participants

This research is part of a wider ongoing study with a longitudinal design that began with 68 participants with and without DLD from the Balearic Islands (Spain), including 41 TD participants and 27 with DLD. Children were drawn from a bilingual linguistic context, in which both Spanish and Catalan languages are fully embedded within the territory, encompassing its social context, policies, and educational systems.

The recruitment process is described in detail in Guirado-Moreno et al. (2025). In summary, it focused on primary education students with persistent language difficulties that affected their communication and learning, and met the CATALISE criteria without other biomedical conditions (Bishop et al., 2016, 2017). The school speech and language teachers filled out a questionnaire covering the children’s sociodemographic background—including a question about the family’s socioeconomic level (low, medium, or high)— medical history, developmental progress in school, and communication and learning history. Participants did not present auditory or visual impairments, autistic traits, attention-deficit disorder, or any mental disability. Students with language difficulties were paired with control peers from the same classroom, who were individually matched for age and gender, and closely aligned in dominant language and socioeconomic background, thereby ensuring a homogeneous comparison group.

Participants were assigned to the DLD or control group based on a combination of school-based referrals and standardized assessments, in line with recommendations to integrate multiple sources of information (including functional observations, caregiver reports, and norm-referenced tests) when identifying language difficulties (Bishop et al., 2016). Following CATALISE guidelines (Bishop et al., 2016, 2017), a flexible cut-off below the 25th percentile on the Core Language Score (CLS) of the Clinical Evaluation of Language Fundamentals-4 Spanish Edition (CELF-4; Semel, Wigg & Secord, 2006) was used to include children with persistent, functionally impactful language difficulties, along with an IQ standard score greater than 75 in the Raven’s Progressive Matrices Test (Raven, Court & Raven, 1995). The Spanish version of CELF-4 is considered a reliable and valid instrument for assessing Spanish language skills and is commonly used in DLD research in Spanish-Catalan bilingual contexts (see, for example, Ahufinger et al., 2022, 2021; Giberga et al., 2025). The 25th percentile in the CLS was chosen because it reflects performance meaningfully below the normative mean while maintaining sensitivity to functional language impairments that may not reach stringent thresholds. This criterion was considered particularly appropriate for identifying children who experienced difficulties affecting academic and everyday functioning, rather than capturing only the most severe cases. Control participants showed typical development according to school reports and scored above the 25th percentile on both language and nonverbal IQ measures, difficulties affecting academic and everyday functioning, rather than capturing only the most severe cases. Control participants showed typical development according to school reports and scored above the 25th percentile on both language and nonverbal IQ measures.

The final sample comprised 38 children who met all criteria for their respective groups (DLD, Control) and had data available for all three evaluation waves. Thirty participants were excluded for not meeting these requirements: five did not meet the inclusion criteria for DLD (CLS percentiles ranged from 42 to 75), and 25 were excluded due to missing data. The elevated attrition rate was likely associated with the study’s three-wave longitudinal design and the timing of data collection during the COVID-19 pandemic. Table 1 provides a summary of the primary demographic characteristics, categorized by group.

**Table 1:** Descriptive data of the participant’s sociodemographic characteristics, divided by group.

	Control (*n* = 26)	DLD (*n* = 12)	Statistic (*p*)
**Sex**			
Girls	12	5	χ^2^ (1, *N* = 38) = 0.07 (0.796)
Boys	14	7	
**SES** *			
Low	0	0	χ^2^ (2, *N* = 38) = 3.83 (0.148)
Medium	21	10	
High	3	1	
	**Mean (*SD*)**		
**Age** (years)			
Wave 1	11.37 (1.14)	11.66 (0.71)	*t* = 0.81 (0.423)
**CLS** (percentile)	64.27 (12.57)	16.83 (4.45)	*t* = 12.64 (<0.001)
**NV IQ**	96.08 (12.59)	102.25 (15.09)	*t* = −1.32 (0.195)

**Notes:**

SD, Standard deviation; SES, socioeconomic status; CLS, core language score of CELF-IV; NV IQ, non-verbal intellectual quotient with Raven’s test.

* Two missing data in SES in the control group and one missing data in the DLD group.

### Instruments

To assess visuospatial WM, the computerized Backward Corsi Task (BCT) included in the PEBL platform was used (Mueller & Piper, 2014). This task evaluates participants’ capacity to recall in reverse order the sequence in which visual stimuli are presented. Nine blue squares on the monitor alter their colours in a variable order, and participants are required to reproduce this colour-changing sequence in reverse. The length of the sequence gradually increases, starting with two squares. After completing two sequences of a particular length (with at least one correct response), the task immediately progresses to the next length, adding one more square to the sequence. The test stops when the participant is unable to correctly recall the order, making two errors in a sequence of a specified length.

The BCT is considered a measure of visuospatial WM because it not only requires the storage of visual sequences but also their manipulation (reversing the order), thus engaging central executive (CE) resources. CE tasks involve continuous updating and manipulation of information (Henry & Botting, 2017).

### Procedure

This longitudinal design was composed of three evaluative waves. Two years elapsed between the first (mean age = 11.46 years, *SD* = 1.03) and the second wave (mean age = 13.84 years, *SD* = 0.98), due to the timing of the COVID-19 pandemic. In contrast, one year elapsed between the second and the third wave (mean age of 14.47 years, *SD* = 0.95). While visuospatial WM (BCT) was assessed across all waves, both the language ability (CLS from the CELF-IV’s subtests) and cognitive ability (Raven’s Progressive Matrices Test) were evaluated one year before the first wave to assign participants to each group. Our team individually administered all instruments at the schools, ensuring the tests were conducted under optimal conditions.

The University of the Balearic Islands research ethics committee approved this study and obtained full consent (304CER22 is the IRB approval number). Furthermore, all parents of participating students signed informed written consent at the outset of the study. They were also informed of their right to withdraw their child from the study at any time.

### Data analysis

Statistical analyses were performed utilizing JASP (Version 0.18.3; JASP Team, 2024). A significance boundary of *p* < 0.05 was adopted for all analyses. For preliminary demographic comparisons, involving sex and socioeconomic status distributions, the Chi-Square statistic (χ^2^) was used; in the interim, age, language ability, and non-verbal intelligence comparisons were conducted using *t-tests*.

Concerning the BCT, the Total Score measure was used as a dependent variable because it is more sensitive for clinical and experimental ends than the Block Span (Kessels et al., 2000). The Total Score was calculated by multiplying the length of the last correctly recalled pair of sequences (Block Span) by the total number of correctly recalled sequences for each participant. The Shapiro-Wilk test was used to check the normality of the data. Results showed that the Total Score of the BCT adhered to a normal distribution (*p*s > 0.068). Normality was confirmed based on visual inspection of the Q-Q plot. Thus, we proceeded with a 2 (Group: Control, DLD) × 3 (Wave: 1, 2, 3) mixed analysis of variance (ANOVA). Homoscedasticity assumption was not violated as indicated by non-significant results in Levene’s equality of variances tests, all *F*s < 1.03, all *p*s > 0.317. Non-verbal IQ was preliminarily entered as a covariate to explore its potential effect on visuospatial WM. In accordance with Schneider, Avivi-Reich & Mozuraitis (2015), the covariate was centered due to the within-subjects design. No significant main or interaction effects were observed for NV IQ (*p*s > 0.720). Furthermore, the inclusion of this covariate did not meaningfully affect the remaining results. Therefore, it was excluded from further analysis. The *post hoc* pairwise comparison analyses were corrected for multiple comparisons using the Bonferroni adjustments. Greenhouse–Geisser corrections were applied to effects involving repeated measures (Jennings, 1987; Vasey & Thayer, 1987). Finally, *post-hoc* power analyses (G * Power software; v 3.1.9.6.) were conducted for the main and interaction effects of the mixed ANOVA.

## Results

Table 2 presents the main results illustrating the performance in the BCT, divided by wave and group.

**Table 2:** Main outcomes for the BCT by wave and group.

	Control (*n* = 26)		DLD (*n* = 12)	
Wave	*M* (*SD*)	S-W (*p*)	*M* (*SD*)	S-W (*p*)
Wave 1	47.0 (16.3)	0.95 (0.173)	36.1 (11.11)	0.87 (0.068)
Wave 2	56.4 (21.0)	0.94 (0.102)	38.4 (14.6)	0.90 (0.179)
Wave 3	63 (21.4)	0.94 (0.100)	49.3 (22.7)	0.99 (0.148)

**Note:**

DLD, Developmental language disorder; M, mean; SD, standard deviation; S-W, Shapiro-Wilk statistic.

**Figure 1:**
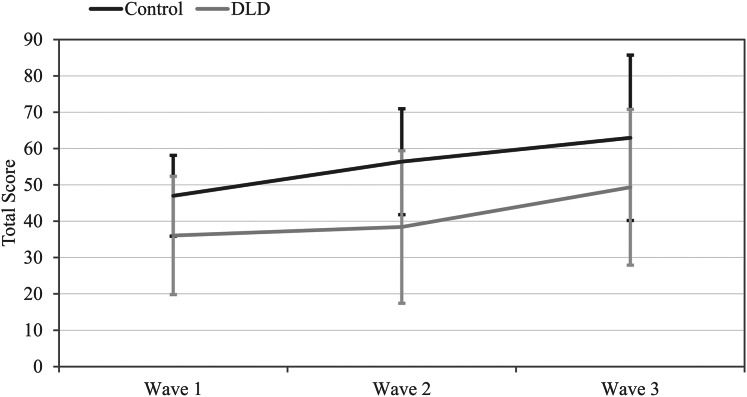
Graphical representation of the BCT outcomes for the two groups in the three waves. Mean total scores on the Backward Corsi Task (BCT) for adolescents with Developmental Language Disorder (DLD) and typically developing (TD) across three waves. Error bars represent standard deviations.

The mixed ANOVA, used to detect differences between groups (Control, DLD) across the three Waves (1, 2, 3) on the BCT, indicated a lower overall BCT Total Score for the group with DLD, as demonstrated by a main Group effect, *F*(1,35) = 7.883, *p* = 0.008, η^2^_p_ = 0.18 (see Fig. 1). The effect size suggests a large effect, with 18% of the variance in BCT performance attributable to group differences (Cohen, 1969). The achieved power for the Group effect was 0.93. In addition, a significant within-subjects main effect of Wave was found, *F*(1.690, 60.857) = 8.005, *p* = 0.001; η^2^_p_ = 0.182, G − G_e_ = 0.845, suggesting a large effect. The achieved power for the Wave effect was 0.99. This finding indicated that the BCT performance improved from Wave 1 to Wave 3, *p* = 0.003, *d* = −0.77. However, this improvement was not revealed between successive waves: between Wave 1 and Wave 2, *d* = −0.31, or between Wave 2 and Wave 3, *d* = −0.46, *p*s > 0.113.

The Group by Wave interaction was not significant, *F*(1.690, 60.857) = 0.689, *p* = 0.596, η^2^_p_ = 0.013, G − G_e_ = 0.845. The achieved power for the interaction was 0.05.

## Discussion

The main objective of this research was to explore the potential differences between adolescents with DLD and their age peers regarding visuospatial WM, taking a developmental perspective. We discuss our results following our hypotheses, anticipating both main effects of Group and Wave as well as an interaction effect.

Our outcomes showed a significant main effect of Group, revealing that adolescents with DLD obtained lower total scores in the visuospatial WM task, confirming our first hypothesis that the DLD group would perform poorer than the age-control group in the visuospatial WM task. This seems to reveal that adolescents with DLD likely experience impairments affecting both visuospatial storage and central executive control. Consequently, this result suggests that deficits in EF in DLD are not limited to verbal tasks and aligns with domain-general hypotheses, explaining that DLD might be defined by a combination of deficits rather than a verbal-specific deficit (Archibald & Gathercole, 2006; Vugs et al., 2013).

The results partially supported our second hypothesis about an improvement in visuospatial WM scores over time in both groups, but particularly in adolescents with DLD. First, we found a main effect of Wave, indicating that both groups improved their scores in the visuospatial WM task across the three waves. This result is in line with other longitudinal research with school-aged children (Blom & Boerma, 2020; Boerma & Blom, 2020) and cross-sectional research comparing children and adolescents (Arslan et al., 2020), suggesting that the development of visuospatial WM in DLD continues throughout childhood into adolescence. However, the Group-by-Wave interaction effect was not found to be significant in our data. Therefore, our results did not provide evidence of differential growth patterns for the DLD and TD groups, as also suggested by the apparently parallel trajectories across waves shown in Fig. 1. However, this interpretation should be treated with caution, given the small sample size and the resulting low statistical power for the Group-by-Wave interaction.

Generally, these results align with some previous studies that found lower ability in the visuospatial WM in school-aged children with DLD. In this vein, comparing our outcomes with studies involving participants of a similar age to our sample indicates that this deficit seems to persist into adolescence. For example, Karasinski & Ellis Weismer (2010), using a proprietary task based on the Odd-One-Out paradigm, found a poorer visuospatial WM capacity in early adolescents with DLD (mean age of 13.92 years) than in their peers. Additionally, the meta-analysis by Vugs et al. (2013) found a lower visuospatial WM in DLD groups than in TD groups, independently of age. Our findings are partially in line with the meta-analysis of Niu et al. (2024), who looked into specific visuospatial WM tasks and reported lower visuospatial WM ability in studies with participants with DLD in the Backward Corsi Task, but they did not report a deficit in the Spatial Span Task among school-aged children, only for the group of preschoolers.

However, our results could be considered contrary to studies suggesting that the difficulties in visuospatial WM are specific to young children with DLD and that these difficulties tend to dissipate as children grow older (Acosta Rodríguez, Ramírez Santana & Hernández Expósito, 2017; Arslan et al., 2020; Everaert et al., 2023; Vugs et al., 2014). For example, Arslan et al. (2020) found no significant evidence for lower visuospatial WM performance in adolescents with DLD (12–18-year-olds) compared to their TD peers, using the same task as the current study. Specifically, Arslan et al. (2020) found that the adolescents with DLD only improved relative to their TD peers in visuospatial WM, but not in other WM dimensions. This result was evidenced by age differences and the correlations between age and visuospatial WM performance, which were stronger in the DLD group than in the TD group (Arslan et al., 2020).

Consequently, our findings partially contradict our expectations, as we hypothesized a greater improvement in the DLD group during the later waves. This suggests that children with DLD may improve as they grow older, but they do so at the same pace as the TD group, even if they start from lower baseline scores in childhood. Our study qualifies the findings of Arslan et al. (2020), who argued that, while children aged 7 to 11 with DLD exhibited impairments in complex processing (measured by backward recall on the Corsi blocks task), the absence of such difficulties in adolescents with DLD suggests a delayed cognitive developmental trajectory. This interpretation aligns with similar reasoning by Hick, Botting & Conti-Ramsden (2005); however, our findings suggest that this developmental delay may persist into early adolescence, suggesting that recovery of visuospatial WM capacity may not have occurred yet. Therefore, our findings may be consistent with previous research indicating that visuospatial WM continues to develop from childhood into adolescence in both groups. Nevertheless, the evidence of difficulties in visuospatial WM in children and adolescents with DLD remains mixed, and no clear pattern emerges.

In this vein, Kapa, Plante & Doubleday (2017) argued that understanding the mixed results in visuospatial WM requires deeper insight into the nature of EF deficits during development. Specifically, as proposed by Vugs et al. (2017), this involves identifying the number and nature of EF components in children with DLD, while also considering the possibility that lower nonverbal intelligence—rather than a specific visuospatial WM impairment—may account for the observed difficulties.

However, in our data, we found that the main group effect was maintained when controlling for nonverbal intelligence, indicating that this factor may not fully account for the observed variability. Similarly, Boerma & Blom (2020) found that differences vanished when verbal short-term memory was controlled for, highlighting the importance of considering multiple cognitive factors. Moreover, Blom & Boerma (2020) found that the severity and persistence of DLD affect working memory, which could explain the discrepancies observed between our data and our hypothesis. In this sense, Acosta Rodríguez, Ramírez Santana & Hernández Expósito (2017) found poorer visuospatial WM only in children with DLD who had severe expressive and receptive impairments, suggesting that visuospatial WM deficits may be associated with greater severity of language impairment. However, despite using a higher cut-off score in the language test in our sample (25^th^ percentile) than the typically used as an inclusion criterion for DLD participants (16^th^ percentile) in past research (Auza, Murata & Peñaloza, 2023; Helo et al., 2025; Krishnan et al., 2021; Suárez-Romón, Martínez López & Suárez-Coalla, 2024), visuospatial WM difficulties seem to persist.

Several factors might explain the lack of Group-by-Wave interaction. For instance, Vugs et al. (2013) found that greater visuospatial storage impairment was associated with more severe language difficulties. In contrast, Arslan et al. (2020), using the same task as the current study, reported significant differences in their group of children, but not in the adolescent group. This discrepancy may be attributed to methodological differences between cross-sectional and longitudinal approaches, with the latter assessing the same participants over time, rather than comparing different age groups. Nonetheless, the limited statistical power in our interaction analysis somewhat constrains the strength of our conclusions regarding the parallel development of visuospatial memory in children with DLD and TD children, underscoring the need for future studies with larger samples to more definitively address this issue.

The present study has several limitations. The most important one is the small sample size, particularly in the DLD group, which is partly inherent to the longitudinal design of the present study. Therefore, the current findings may be non-representative and should be considered preliminary, but they provide an initial and valuable step toward understanding the developmental trajectory of visuospatial WM in children with DLD. Tracking the same individuals over an extended period poses practical challenges, such as participant attrition and the intensive resources required for repeated assessments. This entails a considerable cost in terms of statistical power, which, although adequate for the main effects, is markedly limited with respect to the Group-by-Wave interaction. Another important limitation relates to the use of the Spanish Edition of the CELF-4 (Semel, Wigg & Secord, 2006) for the language assessment of our participants. Although this version was designed to identify language disorder or delay in Spanish-speaking students, its standardization sample was drawn from the Hispanic population in the United States. Therefore, its percentile scores are not norm-referenced for children in Spain and should be interpreted with caution. However, because there is currently no more suitable instrument for assessing language abilities in Spanish or Spanish–Catalan children, the Spanish Edition of the CELF-4 (Semel, Wigg & Secord, 2006) remains the most commonly used standardized tool in similar Catalan-Spanish studies (*e.g*., Ahufinger et al., 2021, 2022; Giberga et al., 2025), allowing for comparability across research.

Moreover, the limited range of tasks used to assess visuospatial working memory (WM) is another limitation. Incorporating a broader set of visuospatial WM measures would help mitigate task-specific biases and enhance the reliability and consistency of the findings. At the same time, the diversity of tasks used in the literature to assess visuospatial WM, with differences in complexity, modality, and processing demands, hinders the comparison of conclusions across studies. Additionally, recalling more than three items increases cognitive load and recruits central executive resources (Vandierendonck et al., 2004). This indicates that the task may engage broader cognitive processes, such as inhibitory control, complicating a straightforward interpretation of performance as a measure of pure visuospatial ability. Such complexity is typical of tasks with high cognitive demands (Larson et al., 2023). Future research would benefit from incorporating visuospatial WM tasks with lower executive requirements—such as the forward Corsi task—to better isolate storage-specific components of working memory. Finally, a minor limitation of the present study is that the intervals between assessment waves were not uniform (2 years between Wave 1 and Wave 2, and 1 year between Wave 2 and Wave 3). Nevertheless, considering the effect sizes, the longer interval does not appear to result in a greater increase in task performance.

## Conclusions

In conclusion, the present study offers preliminary but valuable results about early-emerging deficits in working memory among primary school children with DLD that seem to persist during adolescence. However, our study also suggests that both groups would exhibit similar overall trends in visuospatial WM development. In addition, these findings emphasize the need for further research to explore underlying mechanisms and potential compensatory strategies that help explain the development of visuospatial WM development among youngsters with DLD. Overall, the present results should be viewed as a call for larger-scale longitudinal replications rather than as a definitive answer regarding the trajectory of visuospatial WM in DLD.

## Supplemental Information

10.7717/peerj.21177/supp-1 Supplemental Information 1 STROBE checklist.

10.7717/peerj.21177/supp-2 Supplemental Information 2 Codebook Data.

## Additional Information and Declarations

### Data Availability

The following information was supplied regarding data availability:

The data available at Zenodo and OSF:

- Guirado Moreno, J. L., Aguilar-Mediavilla, E., Esteller-Cano, A., López-Penadés, R., & Adrover-Roig, D. (2025). Examining longitudinal changes in visuospatial working memory in adolescents with Developmental Language Disorder [Data set]. Zenodo. https://doi.org/10.5281/zenodo.17629924

- https://osf.io/raf26/overview.
