# Supplementary material for: Examining longitudinal changes in visuospatial working memory in adolescents with Developmental Language Disorder
Source: PeerJ. 2026 Apr 23;14:e21177. doi: 10.7717/peerj.21177 (PMC13110649; doi:10.7717/peerj.21177)
Supplement: Supplemental Information 1 [file peerj-14-21177-s001.docx]

# STROBE Statement—Checklist of items that should be included in reports of observational studies

| Item No | Recommendation | Location in the manuscript | Notes |
| --- | --- | --- | --- |
| 1a | Indicate the study’s design with a commonly used term in the title or the abstract | Title (p.1), Abstract (p.2) | Longitudinal design clearly stated |
| 1b | Provide in the abstract an informative and balanced summary of what was done and what was found | Abstract (p.2) | Done |
| 2 | Explain the scientific background and rationale for the investigation | Introduction (pp. 3–7) | Extensive background and rationale provided |
| 3 | State specific objectives, including any prespecified hypotheses | Introduction (end, p. 7) | Two hypotheses clearly stated |
| 4 | Present key elements of study design early in the paper | Methods – Procedure (pp, end, p 8–11) | Longitudinal design, 3 waves |
| 5 | Describe the setting, locations, and relevant dates, including periods of recruitment, exposure, follow-up, and data collection | Methods – Participants & Procedure (pp, end, p 7–9) | Recruitment in Balearic Islands; waves and COVID delay described |
| 6a | Give the eligibility criteria, and the sources and methods of selection of participants. Describe methods of follow-up | Methods – Participants (pp. end, 7–9) | Eligibility, inclusion/exclusion, follow-up explained |
| 6b | For matched studies, give matching criteria and number of exposed and unexposed | Methods – Participants (p. 8) | Controls matched by age, gender, SES; n=12 DLD, n=26 TD |
| 7 | Clearly define all outcomes, exposures, predictors, potential confounders, and effect modifiers | Methods – Instruments (p. 9) | Outcome = visuospatial WM (BCT); confounder NV IQ tested |
| 8 | For each variable of interest, give sources of data and details of methods of assessment (measurement) | Methods – Instruments (p. 9) | BCT task, CELF-IV, Raven described |
| 9 | Describe any efforts to address potential sources of bias | Methods – Participants (p. 8); Data analysis (p. 11) | Matched controls; IQ covariate tested |
| 10 | Explain how the study size was arrived at | Methods – Participants (pp, end p 7 – 11) | Final n=38; no power calculation reported |
| 11 | Explain how quantitative variables were handled in the analyses | Methods – Data analysis (pp. 10–11) | BCT total score explained; ANOVA approach used |
| 12a | Describe all statistical methods, including those used to control for confounding | Methods – Data analysis (pp. 10–11) | Mixed ANOVA; covariate IQ tested |
| 12b | Describe any methods used to examine subgroups and interactions | Methods – Data analysis (pp. 10–11) | Group × Wave interaction tested |
| 12c | Explain how missing data were addressed | Methods – Participants (p. end p 7-9) | Excluded if incomplete data; no imputation |
| 12d | Explain how loss to follow-up was addressed (cohort study) | Methods – Participants (p. 8) | Only those with 3 waves included |
| 12e | Describe any sensitivity analyses | Not reported | No sensitivity analyses performed |
| 13a | Report numbers of individuals at each stage of study | Methods – Participants (p. 8) | n=68 initial; n=38 final sample |
| 13b | Give reasons for non-participation at each stage | Methods – Participants (p. 8) | Excluded for missing data or not meeting criteria |
| 13c | Consider use of a flow diagram | Not included |  |
| 14a | Give characteristics of study participants | Methods – Participants (Table 1, p. 9, but tha table is included in the supplementary files) | Demographic characteristics reported |
| 14b | Indicate number of participants with missing data for each variable | Not explicitly reported | Only complete cases retained |
| 14c | Summarise follow-up time | Methods – Procedure (p. 10) | Follow-up intervals described |
| 15 | Report numbers of outcome events or summary measures over time | Results – Table 2, Figure 1 (pp. 11–12) | BCT scores per group and wave reported |
| 16a | Give unadjusted and adjusted estimates with precision | Results (pp. 11–12) | Group and Wave effects with F, p, η² reported; adjusted IQ tested |
| 16b | Report category boundaries when continuous variables were categorized | Not applicable | No categorization performed |
| 16c | If relevant, consider translating relative risk into absolute risk | Not applicable | Not a risk study |
| 17 | Report other analyses done | Results (p. 12) | Covariate IQ tested |
| 18 | Summarise key results with reference to study objectives | Discussion (pp. 12–15) | Objectives revisited; hypotheses addressed |
| 19 | Discuss limitations of the study | Discussion (p. 16) | Sample size, task limitations discussed |
| 20 | Give a cautious overall interpretation considering objectives, limitations, multiplicity of analyses, results from similar studies, and other relevant evidence | Discussion (pp. 12–16) | Interpretation balanced with prior literature |
| 21 | Discuss the generalisability (external validity) of the study results | Discussion (pp. 12–16) | Limitations and scope discussed |
| 22 | Give the source of funding and the role of the funders | Acknowledgments (p. 17) | Funding sources reported; no role of funders indicated |
