# Supplementary material for: Examining longitudinal changes in visuospatial working memory in adolescents with Developmental Language Disorder
Source: PeerJ. 2026 Apr 23;14:e21177. doi: 10.7717/peerj.21177 (PMC13110649; doi:10.7717/peerj.21177)
Supplement: Supplemental Information 2 [file peerj-14-21177-s002.docx]

# CODEBOOK

***Gender****: 1= Male ; 0= Female*

***Group_Rev_corsi****: CON= Included Control group DLD=Included in DLD group*

***CLS_SS_rev2_def:*** *standard Score of Core Language of CELF-IV*

***CLS_PC_rev2_def:*** *Percentile of Core Language of CELF-IV*

***RV_CI:*** *Standard Score of CI of Raven's Test*

***SD_SES:*** *Socioeconomic level of the participant’s family: Bajo= low Medio= medium Alto= high*

***Corsi_MO2_total score:*** *Product of the length of the last correctly remembered pair of sequences (block span) and the total number of correctly remembered sequences in Wave 1.*

***Corsi_MO4_total score:*** *Product of the length of the last correctly remembered pair of sequences (block span) and the total number of correctly remembered sequences in Wave 2.*

***Corsi_MO5_total score:*** *Product of the length of the last correctly remembered pair of sequences (block span) and the total number of correctly remembered sequences in Wave 3.*
